# Increased and unjustified CT usage in paediatric C-spine clearance in a level 2 trauma centre

**DOI:** 10.1007/s00068-020-01520-z

**Published:** 2020-10-27

**Authors:** Joost G. ten Brinke, Geertruida Slinger, Annelie Slaar, Teun Peter Saltzherr, Mike Hogervorst, J. Carel Goslings

**Affiliations:** 1 Department of Surgery, Gelre Hospital, Apeldoorn, The Netherlands; 2 Trauma Unit, Department of Surgery, Amsterdam UMC, University of Amsterdam, Meibergdreef 9, 1105 Amsterdam, The Netherlands; 3 Department of Radiology, Dijklander Ziekenhuis, Hoorn, The Netherlands; 4 Department of Surgery, Haaglanden MC, The Hague, The Netherlands; 5 Department of Trauma Surgery, Onze Lieve Vrouwe Gasthuis, Amsterdam, The Netherlands

**Keywords:** Paediatric, Cervical spine injury, Trauma protocols, Radiographic imaging, CT usage

## Abstract

**Purpose:**

Cervical spine injury after blunt trauma in children is rare but can have severe consequences. Clear protocols for diagnostic workup are, therefore, needed, but currently not available. As a step in developing such a protocol, we determined the incidence of cervical spine injury and the degree of protocol adherence at our level 2 trauma centre.

**Methods:**

We analysed data from all patients aged < 16 years suspected of cervical spine injury after blunt trauma who had presented to our hospital during two periods: January 2010 to June 2012, and January 2017 to June 2019. In the intervening period, the imaging protocol for diagnostic workup was updated. Outcomes were the incidence of cervical spine injury and protocol adherence in terms of the indication for imaging and the type of imaging.

**Results:**

We included 170 children in the first study period and 83 in the second. One patient was diagnosed with cervical spine injury. Protocol adherence regarding the indication for imaging was > 80% in both periods. Adherence regarding the imaging type decreased over time, with 45.8% of the patients receiving a primary CT scan in the second study period versus 2.9% in the first.

**Conclusion:**

Radiographic imaging is frequently performed when clearing the paediatric cervical spine, although cervical spine injury is rare. Particularly CT scan usage has wrongly been emerging over time. Stricter adherence to current protocols could limit overuse of radiographic imaging, but ultimately there is a need for an accurate rule predicting which children really are at risk of injury.

## Introduction

Paediatric patients suffering blunt trauma are always assessed for cervical spine injury (CSI) given the potential risk of morbidity and mortality [1–3]. However, CSI is rare, accounting for less than 2% of all children being evaluated after blunt trauma, even in higher risk populations presenting at level 1 trauma centres [4–7]. Identifying children at risk of CSI is challenging, since physical examination can be unreliable [8]. In addition, trauma mechanisms in children differ from those in adults and are often associated with unique injury patterns, due to the anatomy of the paediatric cervical spine (C-spine) [5, 9, 10]. When clearing the C-spine in children, radiographic imaging is often used, despite this being costly and exposing children to radiation and its associated risks. While CSI should not be missed, children at low risk of injury should not be subjected unnecessarily to radiation. If we had a validated clinical decision tool, we could better balance these conflicting interests and predict which children need radiographic imaging. Such a decision tool is, however, currently not available [11].

In the adult population, the National Emergency X-Radiography Utilization Study (NEXUS) criteria and the Canadian C-spine Rule (CCR) have been extensively tested and validated (Tables 1, 2) [12, 13]. Analysis of these tools in children, however, is sparse. A 2017 meta-analysis concluded that the NEXUS criteria are at best a guide to clinical assessment, and not a strict protocol, while evidence for the accuracy of the CCR to detect CSI in the paediatric population is lacking [14]. Nevertheless, current international guidelines recommend combining both predicting rules [11, 15, 16].

**Table 1:** Features of the NEXUS criteria [12]

Midline tenderness of the C-spine
Focal neurologic deficit
Altered level of consciousness^a^
Evidence of intoxication
Distracting injuries^b^

*C-spine* cervical spine

^a^Defined as a score of < 15 on the Glasgow Coma Scale (GCS)

^b^Including fractures of long bones, visceral injury, crush or laceration, burns

**Table 2:** Features of the CCR [13]

Age ≥ 65 years
Paraesthesia in the extremities
Dangerous trauma mechanism (DTM)
Fall from ≥ 0.9 m
Axial load injury (e.g., diving)
High speed motor vehicle accident (> 100 km/hr, rollover or ejection)
Bicycle collision
Accident with motorized recreational vehicle
Inability to actively rotate the neck > 45°

If CSI is suspected, various international trauma guidelines recommend plain radiography of the C-spine as primary imaging in children [11, 16]. Its sensitivity for detecting CSI is higher than 90% [9, 11, 17–19]. A CT scan of the C-spine is only indicated in those patients for whom a fracture is seen on plain radiography or for whom there is clinical suspicion of CSI despite a negative result with plain radiography [20–22]. A CT scan of the C-spine is the primary imaging modality exclusively in patients who are haemodynamically unstable or who have a reduced level of consciousness [23, 24]. The main reason for this is the increased risk of thyroid cancer: the relative risk from a CT scan is thought to be 13–25% higher than the risk from a plain radiograph [21, 25, 26], except for low dose CT.

A first step towards developing a validated clinical decision rule is to determine the size of the problem and evaluate current practice. This is particularly crucial in low-risk populations, since radiographic imaging will have only minimal therapeutic yield in this group. We, therefore, analysed CSI clearance in our level 2 trauma centre during two periods, whereby our local protocol was updated and re-implemented in the intervening years. Our specific research questions were: (I) What is the incidence of CSI in the paediatric population at our level 2 trauma centre? (II) What is the degree of protocol adherence regarding when and what type of imaging should be requested at our level II trauma centre?

## Methods

### Study design and population

We conducted a retrospective cohort study that included all children under the age of 16 presenting at the emergency department (ED) of a Dutch level 2 trauma centre and large teaching hospital with suspected CSI after blunt trauma, for whom radiography of the C-spine had been obtained. Two study periods were defined; one before and one after the re-implementation of the updated protocol in 2015. The first period (P1) was from January 2010 to June 2012 and the second period (P2) was from January 2017 to June 2019. Data were extracted from the computerized database of the hospital's radiology department and from electronic medical records.

### Protocols and implementation strategy

**Fig. 1:**
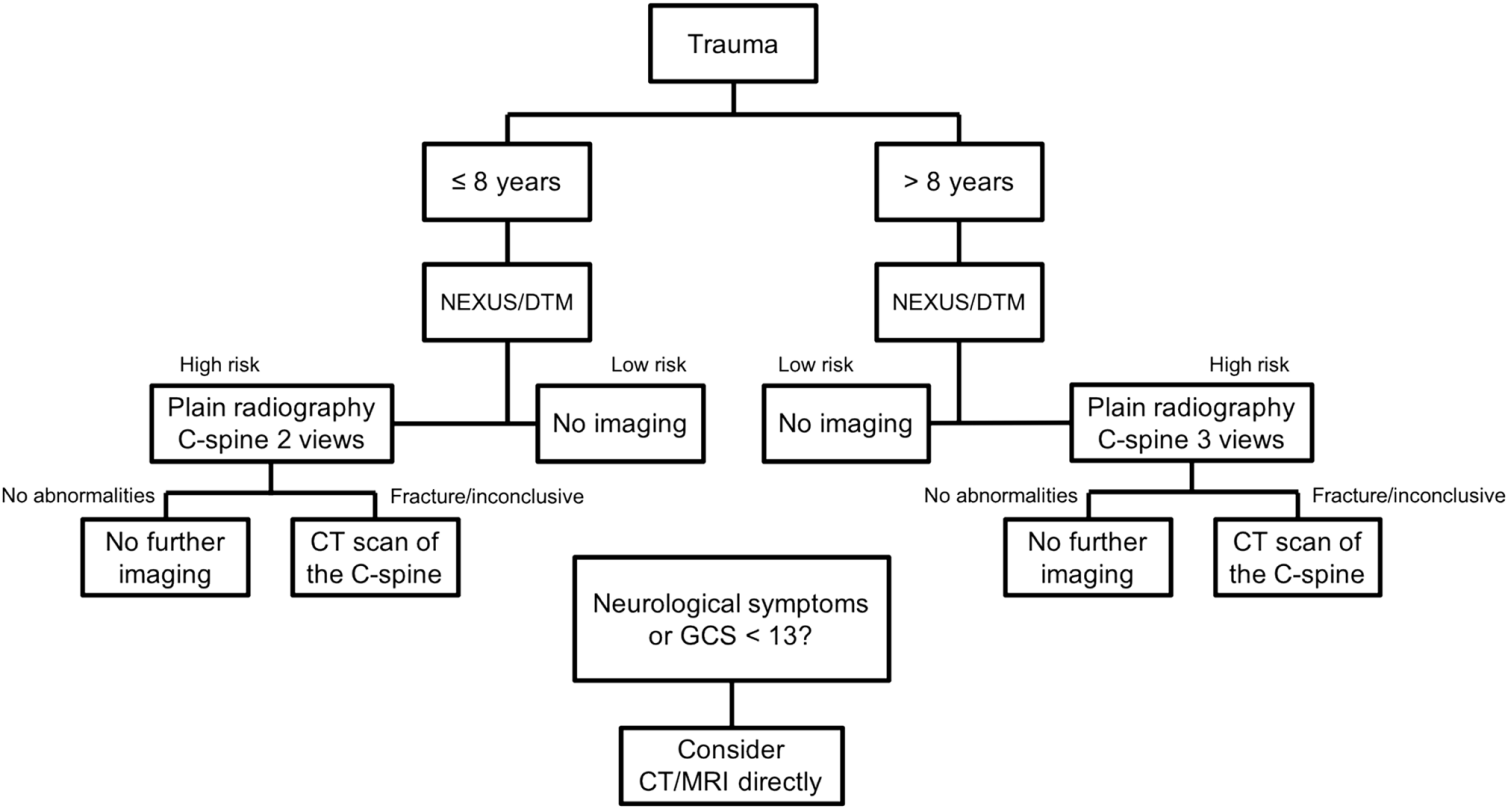
Flowchart of trauma imaging protocol used in study period 1, implemented in 2010. *NEXUS* National Emergency X-Radiography Utilization Study, *DTM* dangerous trauma mechanism, *C-spine* cervical spine, *GCS* Glasgow Coma Scale

The local trauma imaging protocol that was in use during P1 (Fig. 1) had been established through a collaboration between the trauma and radiology departments of our hospital. It was designed for children under the age of 16 and stratified patients by age, discriminating between those up to the age of 8 years and those aged nine and older. According to the protocol, radiographic imaging was required if patients had one or more of the high-risk features listed in the NEXUS criteria or if they had suffered trauma with a dangerous trauma mechanism (DTM), as listed in Table 2. The primary imaging modality was plain radiography. In children where plain radiography was inconclusive or where a fracture was seen, a CT scan of the C­spine was required. A CT scan of the C­spine was recommended as primary imaging modality for patients who had neurological symptoms or a Glasgow Coma Scale (GCS) below 13 at initial assessment.

**Fig. 2:**
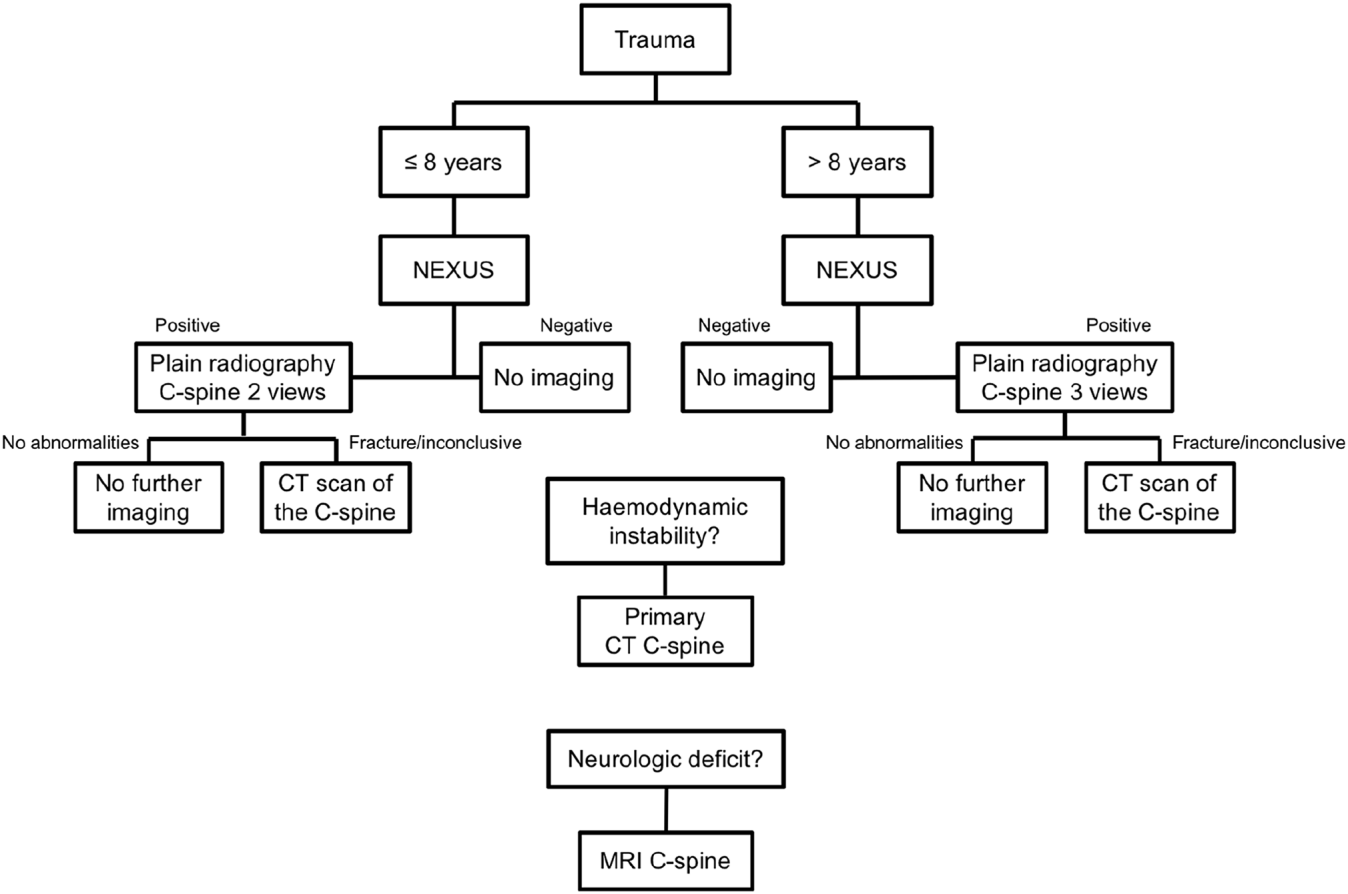
Flowchart of updated trauma imaging protocol used in study period 2, implemented in 2015. *NEXUS* National Emergency X-Radiography Utilization Study, *C-spine* cervical spine

The updated protocol (Fig. 2) used during P2 resembles the initial local protocol but only includes the NEXUS criteria. It also specifies that a CT scan should be used as the primary imaging modality for children who are haemodynamically unstable.

When implementing the updated protocol, we took account of factors previously reported to promote effective improvement of protocols in patients care [27]. In practice, this involved the following three components: (1) one trauma surgeon was responsible for preventing unnecessary imaging and for distributing the updated protocol; (2) all doctors and residents of the surgery, orthopaedic, and radiology departments at our hospital were informed about the protocol by e-mail and through presentations at in-hospital meetings; and (3) regional general practitioners were informed about the updated protocol, since we had noticed that they also frequently ordered imaging of the C-spine after trauma.

### Definitions and outcome parameters

CSI was defined as any fracture or dislocation of the C-spine. CSI also included any neurologic deficit—comprising any new shortcoming in motor or sensory functioning—caused by blunt trauma of the C-spine. A DTM was defined in accordance with the CCR definition (Table 2) [13]. We assessed the study populations from both periods for the following outcomes: incidence of CSI; frequency of plain radiography of the C-spine as primary imaging modality; frequency of CT of the C-spine as primary and as additional imaging modality; and adherence to the protocol with regard to (1) the indication for radiographic imaging and (2) the type of radiographic imaging.

### Statistical analysis

All data were analysed with R statistical software version 3.5.3 (R Foundation for Statistical Computing, Vienna, Austria) using descriptive analyses. In accordance with the radiography protocols, we stratified the analyses in two age categories: (1) 8 years or younger and (2) 9–15 years.

## Results

The 170 children in P1 had a mean age of 9.9 years. The mean injury severity score (ISS) was 5.7. In most patients, the trauma mechanism was a fall from height. The 83 children in P2 had a mean age of 11.5 years. The mean ISS was 2.4. Again, a fall from height was the most prevalent trauma mechanism. All patient characteristics are listed in Table 3.

**Table 3:** Baseline patient characteristics in study periods 1 and 2

	P1 (2010–2012)	P2 (2017–2019)
Total included, *n*	170	83
Male sex, *n* (%)	96 (56.5)	39 (47.0)
Mean age, yrs	9.9	11.5
Children ≤ 8 yrs, *n* (%)	53 (31.2)	17 (20.5)
Children > 8 yrs, *n* (%)	117 (68.8)	66 (79.5)
Hospitalised, *n* (%)	74 (43.5)	21 (25.3)
Mean duration of admission, days	2.2	2.1
Mean ISS, score (1–75)	5.7	2.4
Trauma mechanism		
Fall from height < 2.5 m, *n* (%)	44 (25.9)	40 (48.2)
Fall from height > 2.5 m, *n* (%)	45 (26.5)	5 (6.1)
Bicycle or horse accident, *n* (%)	37 (21.8)	18 (21.7)
Motor vehicle accident, *n* (%)	22 (12.9)	8 (9.6)
Cyclist or pedestrian versus car, *n* (%)	15 (8.8)	6 (7.2)
Person versus object, *n* (%)	7 (4.1)	6 (7.2)

*n* number, *yrs* years, *ISS* Injury Severity Score

### Incidence of CSI

In the first study period, no CSI was diagnosed after the initial work-up at the ED. Also, none of the patients were readmitted to our hospital with a missed injury. The incidence of CSI in P1 was, therefore, zero.

In the second study period, one patient was diagnosed with CSI: a fracture of vertebral body C5, without ligamentous injury. This patient was successfully treated with a hard collar for 6 weeks (Table 4). This one patient with CSI diagnosed gave an incidence in P2 of 1.2%. To our knowledge, no CSI was missed at first presentation at the ED. For all patients taken together (P1 and P2), the incidence rate of CSI was 0.40%.

**Table 4:** Characteristics of the patient found to have cervical spine injury

Period	Age	Sex	MOI	ISS	NEXUS / DTM	Spinal injury	Initial radiography	Treatment	Other injuries	Radiological FU
P2	13	F	Horse accident	5	+ / +	Ventral fracture body C5, ligaments intact	C-spine CT scan + subsequent MRI	Hard collar for 6 weeks	Excoriations extremities	3 C-spine X-rays

*P2* study period 2, *MOI* mechanism of injury, *ISS* Injury Severity Score, *NEXUS* National Emergency X-Radiography Utilization Study, *DTM* dangerous trauma mechanism, *FU* follow-up, *C-spine* cervical spine

### Radiography

In P1, a plain radiograph of the C­spine was the primary diagnostic imaging modality in 165/170 patients (97.1%). When we stratified the patients into groups based on age, the percentages of plain radiography as primary imaging modality were similar in the two age groups. A CT scan of the C-spine was the primary diagnostic imaging modality in 5/170 patients (2.9%). An additional CT scan, after plain radiography, was performed in 22/165 patients (13.3%). All of those scans were without abnormalities. A higher proportion of additional CT scans was performed in children above the age of 8 (Table 5). No MRI scans were obtained, neither as primary nor as additional imaging modality.

**Table 5:** Radiography and NEXUS criteria in study periods 1 and 2

	P1 (2010–2012)	P2 (2017–2019)
Radiography obtained in children ≤ 8 yrs, *n* (%)	53/170 (31.2)	17/83 (20.5)
Initial plain radiography (X-rays), *n* (%)	51/53 (96.2)	15/17 (88.2)
Additional CT scans, *n* (%)	2/51 (3.9)	1/15 (6.7)
Initial CT scans, *n* (%)	2/53 (3.8)	2/17 (11.8)
Radiography obtained in children > 8 yrs, *n* (%)	117/170 (68.8)	66/83 (79.5)
Initial plain radiography (X-rays), *n* (%)	114/117 (97.4)	30/66 (45.5)
Additional CT scans, *n* (%)	20/114 (17.5)	2/30 (6.7)
Initial CT scans, *n* (%)	3/117 (2.6)	36/66 (54.5)
Number of patients meeting NEXUS criteria		
0 features (NEXUS negative), *n* (%)	56/170 (32.9)	13/83 (15.7)
Presence of DTM, *n* (%)	38/56 (67.9)	–^a^
Absence of DTM, *n* (%)	18/56 (32.1)	–^a^
1 or more features (NEXUS positive), *n* (%)	114/170 (67.1)	70/83 (84.3)
1 feature, *n* (%)	89/114 (78.1)	58/70 (82.9)
2 features, *n* (%)	18/114 (15.8)	12/70 (17.1)
3 features, *n* (%)	7/114 (6.1)	0/70 (0)
4 features, *n* (%)	0/114 (0)	0/70 (0)
5 features, *n* (%)	0/114 (0)	0/70 (0)

*n* number, *yrs* years, *NEXUS* National Emergency X-Radiography Utilization Study, *DTM* dangerous trauma mechanism

^a^In the adapted protocol used in P2, DTM was no longer a criterion

In P2, plain radiography was the primary imaging modality in 45/83 patients (54.2%). The percentage of children undergoing plain radiography as primary imaging modality was higher in the group aged 8 or younger. A CT scan of the C-spine was the primary diagnostic imaging modality in 38/83 patients (45.8%), with a higher percentage in the group aged 9 and older as compared to the younger group (Table 5). Notably, we observed that many children underwent a combined CT scan of the C-spine and the brain; 72.4% of the children in whom we had obtained a CT scan of the brain received an initial CT scan of the cervical spine. This proportion was higher among the older children (Table 6). After plain radiography, an additional CT scan was performed in 3/45 patients (6.7%); all three scans were negative (Table 5). Overall, one MRI scan of the C-spine was obtained, which was found to be normal (patient characteristics described in Table 4).

**Table 6:** Initial CT scans of the cervical spine and CT scans of the brain in P2

	Primary CT C-spine, *n*	CT brain, *n*	CT brain + primary CT C-spine, *n* (%)
Children ≤ 8 yrs (*n* = 17)	2	9	2 (22.2)
Children > 8 yrs (*n* = 66)	36	20	19 (95.0)

Example: of the 17 children aged 8 or younger, 2 had a primary CT scan of the cervical spine and 9 had a CT scan of the brain. 2 of the children with a CT scan of the brain underwent a primary CT scan of the C-spine (22.2%)

*n* number, *yrs* years, *C-spine* cervical spine

### Protocol adherence

Of the patients in P1, 152/170 (89.4%) met at least one of the NEXUS criteria or had a DTM, which means that they had an indication for radiographic imaging in accordance with the local protocol (Table 5). All of these children received the correct type of primary imaging according to the protocol, whether this was plain radiography or a CT scan. The remaining 18/170 patients (10.6%) were both NEXUS and DTM negative, and, therefore, retrospectively failed to meet one of the criteria for imaging. In this subgroup, no children received an initial or additional CT of the C-spine.

Of the patients in P2, 70/83 (84.3%) met at least one of the NEXUS criteria, thereby justifying radiographic imaging of the C-spine according to the updated protocol. Of all patients in this group, 39 (55.7%) had a primary plain radiography the C-spine and the remaining 31 (44.3%) underwent a primary CT scan. In all 31 children, the reason for an initial CT scan was unclear; one patient had a GCS below 13 but was not haemodynamically unstable. Despite 13/83 patients (15.7%) being NEXUS negative, they nevertheless underwent radiographic imaging. In more than half of them (7/13 patients, 53.8%) a primary CT scan of the C-spine was performed.

## Discussion

While many children presenting at the ED after blunt trauma are suspected of CSI, the actual incidence is low. Adherence to both the initial and updated in-hospital protocol was high in terms of which children required imaging_._ However, in terms of the type of radiographic imaging requested for these children, adherence to the protocol was lower for the updated protocol than for the initial protocol, with much higher CT usage in the second study period.

As mentioned before, CSI incidence among children is low in our hospital, as only one patient with CSI was diagnosed during both study periods. These data correspond with the findings of previous studies—performed both in level 1 trauma centres and other hospitals—which have also reported low incidences. The incidence varies from 0.3–3.7%, depending on the specific population studied [4–7, 28]. Our study confirms that a child presenting at a level 2 trauma centre after blunt trauma is very unlikely to have relevant C-spine injury.

With regard to the indication for imaging, adherence to our initial and updated in-hospital protocol was high. A study by Slaar et al*.* in a level 1 trauma centre also found that Dutch physicians generally do adhere to the guidelines in terms of which children require imaging [4]. Nevertheless, radiologic imaging could have been avoided in at least ten percent of our patients if the protocols had been followed in all cases. We note that there was a substantial difference in the size of the study groups in the two periods, with a reduction from 170 in P1 to 83 in P2. We postulate that the number of children undergoing radiographic imaging has nearly halved since implementing the updated protocol, because physicians are adhering to it more strictly in terms of when imaging is mandatory. Other explanations might be a shift in injury severity, as the ISS and hospitalization rate were both lower in P2 than in P1. Another possibility is a decrease in the number of ED registrations, which we cannot rule out, since we only included patients who underwent radiographic imaging. We consider these explanations unlikely, however, since the out-of-hospital protocols routing patients with suspected CSI did not change in the time between the two study periods. The decrease in hospitalization rate in P2 as compared to P1 could, besides by the decreased ISS, also be declared by the increase in CT usage, which might have given physicians more confidence to send patients home.

In the second study period, a considerable number of patients underwent a primary CT scan without a clear indication. This increased use of CT imaging is an alarming but not isolated phenomenon. Since its introduction, CT usage in general has grown massively, both in adults and in the paediatric population, especially in emergency settings [29–32]. In the paediatric ED, a CT of the brain is by far the most commonly performed CT examination; the largest increase, however, has been reported for CT scans of the cervical spine and chest [32, 33]. And although some studies report that the total volume of CT utilization in children seems to have declined over the last decade—possibly due to the widespread introduction of clinical decision tools [26, 31, 34]—others still report a stable, increased or unexpectedly high CT utilization rate in children suffering blunt trauma [28, 35]. This suggests that protocols are still not being sufficiently implemented in daily practice, or that concurrent developments are hindering physicians’ adherence to these protocols. In this regard, it is possible that our strategy for re-implementing an updated version of the protocol was inadequate, but this is unlikely to fully explain the increase in CT utilization between P1 and P2, since nearly, all primary CT scans performed in P2 would also not have been justified under the previous protocol used in P1. It seems more likely that the protocol in our hospital for brain scanning is largely responsible, since the majority of children, particularly the older ones, who underwent a CT scan of the brain also had a CT scan of the C-spine as primary imaging modality. Combining imaging of the brain and spine in a single radiographic examination might be time efficient but cannot be justified. Furthermore, many physicians still underestimate the amount of radiation exposure due to CT imaging and the associated cancer risks, as shown by previous studies [36–40]. This underestimation might be resulting in physicians using adult standards in the diagnostic work-up of trauma in older children.

This study has a number of limitations. A retrospective study design is inherently associated with the risks of information bias and selection bias. A second limitation is that we evaluated protocol adherence in a level 2 trauma centre, while patients with more severe trauma and a higher chance of CSI are more likely to present to level 1 centres. However, evaluation of CSI clearance in level 2 centres is nevertheless relevant, especially given the apparent overuse of radiographic imaging in a population that has relatively mild trauma and, therefore, a relatively low risk of CSI. A third limitation lies in the fact that we only included patients who had undergone radiographic imaging, which means that we might have missed patients who had CSI but did not undergo radiographic imaging. Consequently, we cannot know whether or not these patients met one of the imaging criteria and might have overestimated protocol adherence regarding which children required imaging. To rule out any such missed injuries, we actively searched for secondary hospital visits in patients records. However, we did not monitor follow-up visits at other hospitals, so we may have underestimated the incidence of CSI at our hospital.

This study shows daily practice and adherence to imaging protocols. It also illustrates the need for developing and validating a clinical decision tool for clearing the C-spine of injury in children. Since evidence for the application of the NEXUS criteria in the paediatric population is sparse, there is a need for prospective research to evaluate its diagnostic accuracy or to find other predictors of CSI. The need for such research is particularly highlighted by our observation that almost all children being NEXUS positive do not have CSI. This means that, under the current protocol, even 100% adherence leads to radiographic overuse. Given the current level of evidence, we respectfully disagree with Hale et al*.* who have recommended primary CT imaging in the evaluation of suspected CSI in children, even when taking into account that they studied a level 1 trauma centre population [41]. CT usage in the paediatric population should be avoided where possible to limit its adverse effects and unnecessary health care costs. The advent of modern new CT scanners of which the radiation dose of a scan of the C-spine is comparable with a conventional C-spine imagine might change this perspective, but until its widespread availability CT use should to be minimized. We also recommend that a combined CT scan of the C-spine and the brain for convenience purposes should meet strict criteria. To limit the use of CT imaging, it is important that all physicians are aware of its strict indications and risks, especially the risk of thyroid cancer. A helpful instrument in improving such awareness is adding a list of imaging criteria as checkboxes to the current CT application form.

## Conclusion

CSI after blunt trauma in a paediatric population of a level 2 trauma centre is rare. Our observation of an increase in CT usage over time—usage that is not in accordance with the local protocol—indicates that improving the benefit-to-risk ratio in the evaluation of cervical spine injury requires stricter adherence to the current protocol. Future studies focused on the development and validation of a better clearance and strategies for adherence are needed to further reduce the risk side of this ratio.
