# Correction to: Increased and unjustified CT usage in paediatric C‑spine clearance in a level 2 trauma centre

**DOI:** 10.1007/s00068-020-01557-0

**Published:** 2021-01-18

**Authors:** Joost G. ten Brinke, Geertruida Slinger, Annelie Slaar, Teun Peter Saltzherr, Mike Hogervorst, J. Carel Goslings

**Affiliations:** 1 Department of Surgery, Gelre Hospital, Apeldoorn, The Netherlands; 2 Trauma Unit, Department of Surgery, Amsterdam UMC, University of Amsterdam, Meibergdreef 9, 1105 Amsterdam, The Netherlands; 3 Department of Radiology, Dijklander Ziekenhuis, Hoorn, The Netherlands; 4 Department of Surgery, Haaglanden MC, The Hague, The Netherlands; 5 Department of Trauma Surgery, Onze Lieve Vrouwe Gasthuis, Amsterdam, The Netherlands

**Correction to: European Journal of Trauma and Emergency Surgery **10.1007/s00068-020-01520-z

The original version of this article unfortunately contained mistakes.

The presentation of Fig. 1 and 2 and the legend of Fig. 2 were incorrect. The corrected figures (Figs. 1, 2) and the legend are given below.

**Fig. 1:**
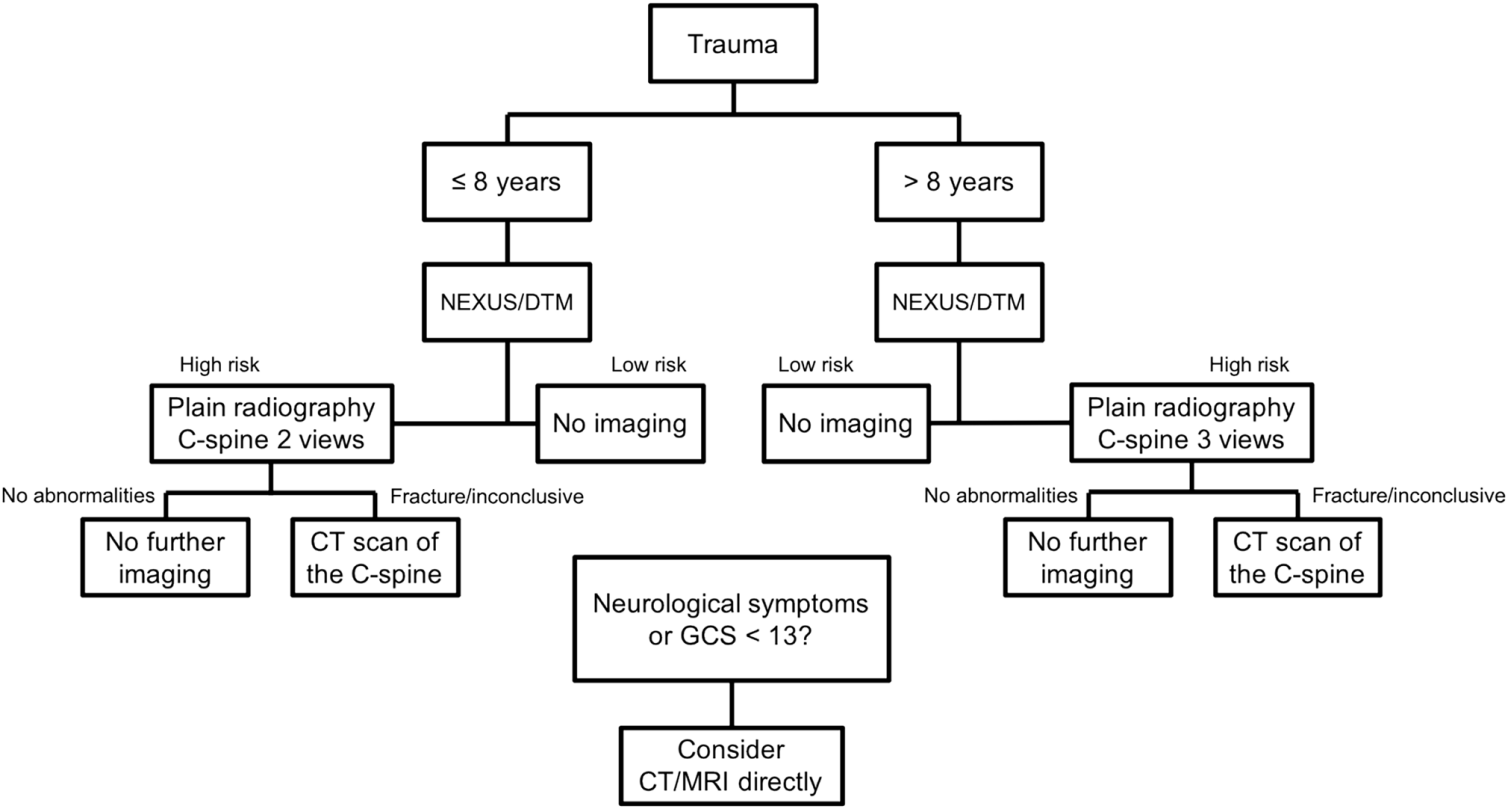
Flowchart of trauma imaging protocol used in study period 1, implemented in 2010. *NEXUS* National Emergency X-Radiography Utilization Study, *DTM* dangerous trauma mechanism, *C-spine* cervical spine, *GCS* Glasgow Coma Scale

**Fig. 2:**
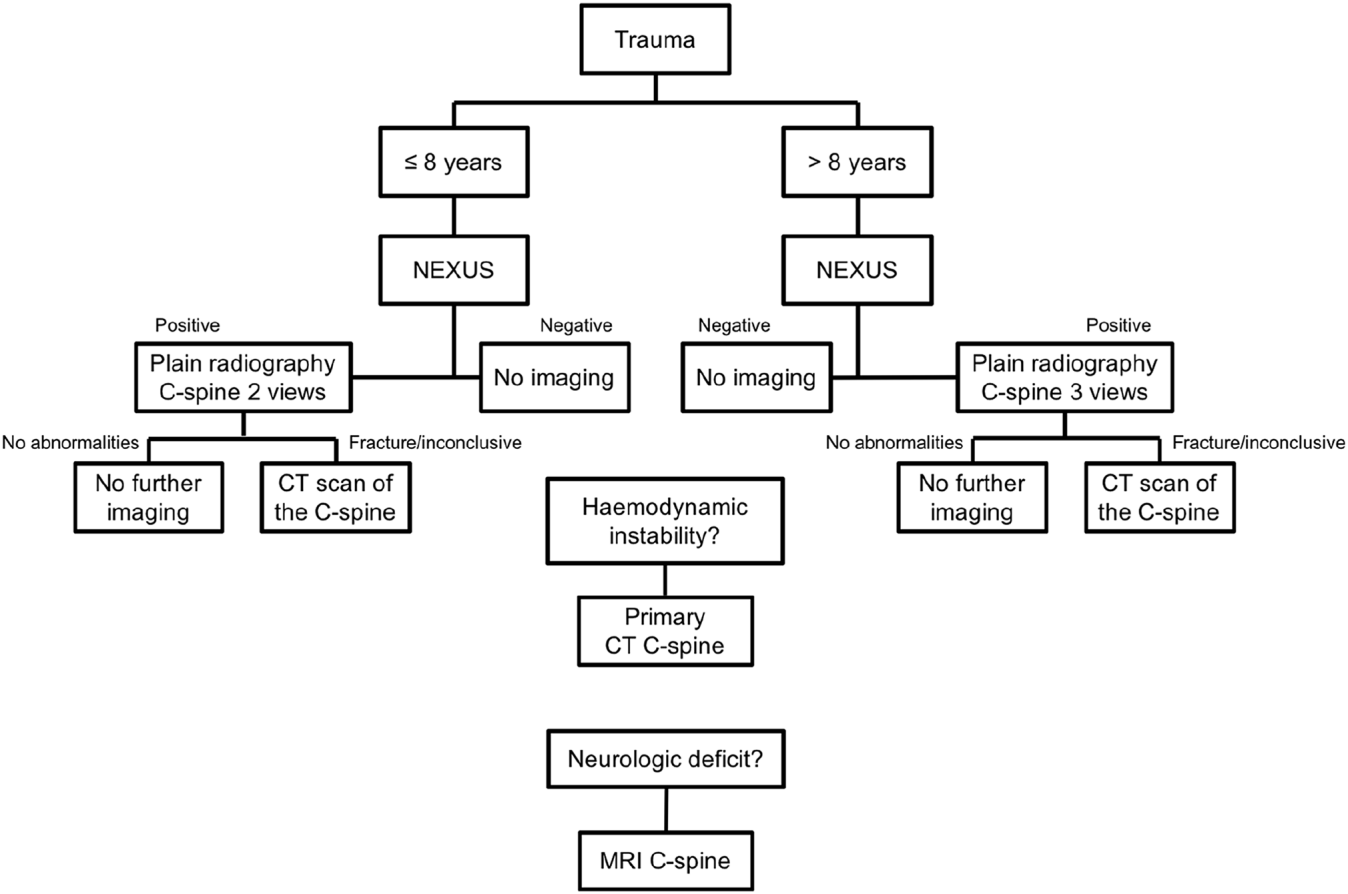
Flowchart of updated trauma imaging protocol used in study period 2, implemented in 2015. *NEXUS* National Emergency X-Radiography Utilization Study, *C-spine* cervical spine

Further the titles of table 4 and 5 and the legend of table 4 were incorrect. The corrected tables (Tables 4, 5) are given below.

**Table 4:** Characteristics of the patient found to have cervical spine injury

Period	Age	Sex	MOI	ISS	NEXUS / DTM	Spinal injury	Initial radiography	Treatment	Other injuries	Radiological FU
P2	13	F	Horse accident	5	+ / +	Ventral fracture body C5, ligaments intact	C-spine CT scan + subsequent MRI	Hard collar for 6 weeks	Excoriations extremities	3 C-spine X-rays

*P2* study period 2, *MOI* mechanism of injury, *ISS* Injury Severity Score, *NEXUS* National Emergency X-Radiography Utilization Study, *DTM* dangerous trauma mechanism, *FU* follow-up, *C-spine* cervical spine

**Table 5:** Radiography and NEXUS criteria in study periods 1 and 2

	P1 (2010–2012)	P2 (2017–2019)
Radiography obtained in children ≤ 8 yrs, *n* (%)	53/170 (31.2)	17/83 (20.5)
Initial plain radiography (X-rays), *n* (%)	51/53 (96.2)	15/17 (88.2)
Additional CT scans, *n* (%)	2/51 (3.9)	1/15 (6.7)
Initial CT scans, *n* (%)	2/53 (3.8)	2/17 (11.8)
Radiography obtained in children > 8 yrs, *n* (%)	117/170 (68.8)	66/83 (79.5)
Initial plain radiography (X-rays), *n* (%)	114/117 (97.4)	30/66 (45.5)
Additional CT scans, *n* (%)	20/114 (17.5)	2/30 (6.7)
Initial CT scans, *n* (%)	3/117 (2.6)	36/66 (54.5)
Number of patients meeting NEXUS criteria		
0 features (NEXUS negative), *n* (%)	56/170 (32.9)	13/83 (15.7)
Presence of DTM, *n* (%)	38/56 (67.9)	–^a^
Absence of DTM, *n* (%)	18/56 (32.1)	–^a^
1 or more features (NEXUS positive), *n* (%)	114/170 (67.1)	70/83 (84.3)
1 feature, *n* (%)	89/114 (78.1)	58/70 (82.9)
2 features, *n* (%)	18/114 (15.8)	12/70 (17.1)
3 features, *n* (%)	7/114 (6.1)	0/70 (0)
4 features, *n* (%)	0/114 (0)	0/70 (0)
5 features, *n* (%)	0/114 (0)	0/70 (0)

*n* number, *yrs* years, *NEXUS* National Emergency X-Radiography Utilization Study, *DTM* dangerous trauma mechanism

^a^In the adapted protocol used in P2, DTM was no longer a criterion

The original article has been corrected.

